# Clinical Presentation and Management of Ethoxy-Modified Trisiloxane Surfactant Poisoning

**DOI:** 10.7759/cureus.95116

**Published:** 2025-10-21

**Authors:** Io Chon Vong, Hoi Ip Leong, João Lei

**Affiliations:** 1 Pharmacy Department, Centro Hospitalar Conde de São Januário, Macao, CHN; 2 Emergency Department, Centro Hospitalar Conde de São Januário, Macao, CHN

**Keywords:** adolescent psychiatry, ethoxy-modified trisiloxane, herbicide poisoning, supportive care, surfactant

## Abstract

Suicidal attempts involving herbicide ingestion are extremely rare in Macao. We report the case of a 13-year-old Chinese female student with a history of mental disorder who presented to the emergency department (ED) of Conde S. Januário Hospital (CHCSJ) after intentionally ingesting 30-40 mL of a herbicide containing ethoxy-modified trisiloxane, a compound with limited human toxicity data that warrants clinical attention. On admission, she was asymptomatic and hemodynamically stable, with unremarkable physical examination and laboratory findings. She was admitted for observation. During her stay, she reported only mild abdominal discomfort, which was managed symptomatically. After 20 hours of observation, she remained stable and was discharged with outpatient psychiatric follow-up arranged. This case underscores that even with potentially toxic herbicides, prompt medical assessment and supportive care can lead to a positive outcome, while highlighting the critical need for psychiatric intervention in cases of intentional self-harm.

## Introduction

Herbicides are chemical agents designed to control or eliminate unwanted vegetation. Several classes, including paraquat, glyphosate, organophosphates, and phenoxy compounds (e.g., Agent Orange), are well-documented for their human toxicity, each with distinct clinical manifestations and varying degrees of severity [1]. Ethoxy-modified trisiloxane is a silicone-based surfactant commonly formulated in herbicides to enhance spreading and penetration. While readily available for online purchase in Macao, data on its human toxicity profile following intentional ingestion are scarce. This report describes a case of intentional ingestion of an ethoxy-modified trisiloxane-containing herbicide. By detailing the clinical course and management, we aim to provide a valuable reference for emergency physicians and toxicologists who may encounter similar exposures, thereby contributing to the limited evidence on its human effects.

## Case presentation

In January 2023, a 13-year-old Chinese female student with a documented history of a prior suicidal attempt was brought to the ED of CHCSJ at 16:30 . She had intentionally ingested approximately 30-40 mL of a commercial herbicide containing ethoxy-modified trisiloxane around 14:00 the same day.

On admission, the patient was conscious, alert, and oriented, with a Glasgow Coma Scale score of 15. Vital signs were as follows: blood pressure 138/83 mmHg, heart rate 105 beats per minute, body temperature 37.6°C, and oxygen saturation 100% on room air. Physical examination was unremarkable. Laboratory investigations revealed mild microcytic hypochromic anemia, which was consistent with her pre-existing condition (Table 1). Her metabolic panel, including renal and liver function tests and electrolyte levels, was within normal limits.

**Table 1:** Laboratory results at emergency department admission

Variables	Value	Range
Urea (mmol/L)	2.9	1.8-6.4
Serum creatinine (umol/L)	42	40-68
Glucose (mmol/L)	4.87	4.4-11.1
Sodium (mmol/L)	140	136-145
Potassium (mmol/L)	3.7	3.5-5.1
Chloride (mmol/L)	105	98-107
Calcium (mmol/L)	2.38	2.10-2.55
Phosphorus (mmol/L)	0.96	0.95-1.65
Magnesium (mmol/L)	0.87	0.7-0.91
Albumin (g/L)	45	38-54
Total bilirubin (µmol/L)	4	<15
Aspartate aminotransferase (AST) (U/L)	13	≤32
Alanine aminotransferase (ALT) (U/L)	7	≤33
Red blood cell (×10^12^/L)	5.1	4.0-5.2
Hemoglobin (g/dL)	↓10.4	12.0-16.0
Hematocrit (%)	↓32.4	36.0-47.0
Mean corpuscular volume (fL)	↓63.1	80.0-100.0
Mean corpuscular hemoglobin (pg)	↓20.2	26.0-34.0
Mean corpuscular hemoglobin concentration (g/dL)	32.1	31.0-37.0
Red cell distribution width (%)	↑14.9	11.5-14.5
White blood cell (×10^9^/L)	9.7	4.3-10.0
Neutrophils (×10^9^/L)	6.5	1.9-7.3
Eosinophils (×10^9^/L)	0	0.0-0.7
Basophils (×10^9^/L)	0	0.0-2.0
Lymphocytes (×10^9^/L)	2.6	1.5-4.0
Monocytes (×10^9^/L)	0.5	0.2-0.9
Platelets (×10^9^/L)	270	100-400

As the patient presented 2.5 hours post-ingestion, gastric decontamination procedures were not indicated. Given the absence of a specific antidote for ethoxy-modified trisiloxane, the management strategy focused on vigilant monitoring and supportive care. She was transferred to an observation unit for continuous monitoring.

Over the ensuing 20 hours, the patient reported only mild abdominal pain, which was managed symptomatically with fasting, intravenous crystalloid fluids, and a single 40 mg intravenous dose of esomeprazole. She remained hemodynamically stable throughout the observation period, with no signs of neurological, respiratory, or metabolic deterioration. Following psychiatric assessment and confirmation of clinical stability, she was discharged with an arranged outpatient psychiatric follow-up.

## Discussion

The lack of human poisoning data for ethoxy-modified trisiloxane presents a significant challenge for clinical risk assessment. As a common silicone surfactant in agrochemicals, its primary role is to lower surface tension, thereby enhancing the spreading and wetting of spray solutions on plant surfaces [2]. Surfactants are broadly categorized into anionic, cationic, and nonionic types, with ethoxy-modified trisiloxanes belonging to the nonionic group. Toxicologically, anionic and nonionic surfactants taken orally are of low toxicity according to acute toxicity tests as well as long-term studies, while certain cationics are moderately toxic [3]. Consequently, ingestion of nonionic surfactants like ethoxy-modified trisiloxane is generally associated with mild, self-limiting gastrointestinal irritation, such as nausea, vomiting, or abdominal discomfort, which is entirely consistent with the presentation in this case [4]. Dermal and ocular irritation are also common, and aspiration poses a risk for chemical pneumonitis.

The clinical course observed in our patient was notably less severe than the fulminant, often fatal multi-organ failure characteristic of typical glyphosate-surfactant herbicide poisonings. This stark contrast is vividly illustrated by the recent case series from Chhikara et al. [5], in which all three patients developed life-threatening complications, including refractory metabolic acidosis, hyperkalemia, acute respiratory distress syndrome, and cardiovascular collapse, resulting in two fatalities. The critical determinant of this dramatic divergence in toxicity lies not merely in the presence of a surfactant, but in the specific chemical nature of the surfactant and its synergistic interaction with the active ingredient. In contrast, the ethoxy-modified trisiloxane in the present case demonstrates a far more favorable systemic toxicity profile, accounting for the benign clinical outcome.

The conservative management strategy employed in this case, specifically the decision to forgo gastric lavage and activated charcoal, was clinically prudent and evidence-based. Gastric lavage is generally contraindicated beyond one hour post-ingestion, particularly in a patient without severe symptoms, due to a risk of aspiration that outweighs any potential benefit [6]. Similarly, the efficacy of activated charcoal diminishes significantly after the first hour, rendering its use unwarranted in this scenario, especially for a substance of low systemic toxicity [7].

A paramount consideration in managing such poisonings, which also represents a significant limitation, is the potential presence of undeclared co-formulants. The active surfactant ingredient is often dissolved in organic solvents (e.g., methanol) or other additives not explicitly listed on the product label. These solvents can possess intrinsic toxicities capable of causing more severe complications, such as metabolic acidosis, central nervous system depression, or end-organ damage [8]. Therefore, the benign presentation in this case should not foster complacency; maintaining a high index of suspicion for toxicity from unlisted ingredients is crucial. Management must include an adequate observation period to exclude delayed effects from these potential co-formulants. Given the increasing online availability of such agrochemicals, this case underscores the need for heightened clinician awareness of their variable and potential presentations.

## Conclusions

Intentional ingestion of a herbicide containing ethoxy-modified trisiloxane in this adolescent patient resulted in minimal toxicity, manifesting only as transient abdominal discomfort that resolved with supportive care. This case suggests that the trisiloxane surfactant itself may be of low acute toxicity. However, the absence of a specific antidote necessitates a management approach centered on supportive care and careful clinical monitoring. Crucially, healthcare providers must remain vigilant for potential effects from unlisted toxic solvents or other co-formulants in the commercial product, which could alter the clinical course significantly. The documentation and reporting of similar cases are essential to build a more robust evidence base, which will help to clarify the human toxicity profile of this compound and refine future clinical management guidelines.

## References

[1] Oiseth S, Jones L, Maza E (2025). Herbicide poisoning. https://www.lecturio.com/concepts/herbicide-poisoning/.

[2] (2025). Agricultural silicone surfactant (product brochure). https://www.silfluosilicone.com/uploads/file/silfluo-agricultural-silicone-surfactant.pdf.

[3] Gloxhuber Ch (1974). Toxicological properties of surfactants. Arch Toxicol.

[4] (2025). ASPCApro: Ingredients & Toxicities of Cleaning Products. https://www.aspcapro.org/resource/ingredients-toxicities-cleaning-products.

[5] Chhikara M, Yadav T, Seelwal D (1974). Glyphosate surfactant herbicide poisoning manifestations and management: a case series. Eur J Cardiovasc Med.

[6] Vale JA, Kulig K (2004). Position paper: gastric lavage. J Toxicol Clin Toxicol.

[7] Silberman J, Galuska MA, Taylor A (2025). Activated charcoal. StatPearls.

[8] Bradberry SM, Proudfoot AT, Vale JA (2004). Glyphosate poisoning. Toxicol Rev.

